# Comparative Genomic Analysis of the ICE*Sa*2603 Family ICEs and Spread of *erm*(B)- and *tet*(O)-Carrying Transferable 89K-Subtype ICEs in Swine and Bovine Isolates in China

**DOI:** 10.3389/fmicb.2016.00055

**Published:** 2016-02-02

**Authors:** Jinhu Huang, Yuan Liang, Dawei Guo, Kexin Shang, Lin Ge, Jam Kashif, Liping Wang

**Affiliations:** ^1^College of Veterinary Medicine, Nanjing Agricultural UniversityNanjing, China; ^2^Department of Veterinary Pharmacology, Sindh Agricultural UniversityTandojam, Pakistan

**Keywords:** integrative and conjugative elements, antimicrobial resistance, horizontal gene transfer, ICE*Sa*2603 family, *erm*(B), *tet*(O), *Streptococcus suis*, *Streptococcus agalactiae*

## Abstract

Integrative and conjugative elements (ICEs) of the ICE*Sa*2603 family have been isolated from several species of *Streptococcus* spp.; however, the comparative genomic and evolutionary analyses of these particular ICEs are currently only at their initial stages. By investigating 13 ICEs of the ICE*Sa*2603 family and two ICE*Sa*2603 family-like ICEs derived from diverse hosts and locations, we have determined that ICEs comprised a backbone of 30 identical syntenic core genes and accessory genes that were restricted to the intergenic sites or the 3′-end of the non-conserved domain of core genes to maintain its function. ICE*Sa*2603 family integrase Int_ICE__*Sa*__2603_ specifically recognized a 15-bp *att* sequence (TTATTTAAGAGTAAC) at the 3′-end of *rplL*, which was highly conserved in genus *Streptococcus*. Phylogenetic analyses suggest that extensive recombination/insertion and the occurrence of a hybrid/mosaic in the ICE*Sa*2603 family were responsible for the significant increase in ICE diversity, thereby broadening its host range. Approximately 42.5 and 38.1% of the tested *Streptococcus suis* and *Streptococcus agalactiae* clinical isolates respectively contained ICE*Sa*2603 family Type IV secretion system (T4SS) genes, and 80.5 and 62.5% of which also respectively carried *int*_ICE__*Sa*__2603_, indicating that ICE*Sa*2603 family is widely distributed across these bacteria. Sequencing and conjugation transfer of a novel sequence type ST303 clinical *S. suis* isolate HB1011 demonstrated that the 89K-subtype ICE*Ssu*HB1011 retained its transferrable function, thereby conferring tetracycline and macrolide resistance.

## Introduction

Integrative and conjugative elements (ICEs) are self-transmissible mobile genetic elements (MGEs) that primarily reside in the host cell's chromosome, yet have the ability to be transferred between cells by conjugation (Burrus et al., 2002; Burrus and Waldor, 2004). ICEs are considered as mosaic elements with both phage- and plasmid-like features that can integrate into and replicate with the host cell's chromosomes similar to those observed in bacteriophages, as well as transfers, via conjugation in plasmids. The number of identified ICEs continues to increase with the exponential expansion of sequenced complete genomes (Te Poele et al., 2008; Wozniak and Waldor, 2010). Guglielmini et al (Guglielmini et al., 2011) previously described the prevalence and diversity of ICEs by conducting bioinformatics analysis of clustered conjugative apparatus modules in various chromosomal locations. Based on this definition, 18% of sequenced prokaryotic genomes contain at least one ICE, implying that the role of ICEs in horizontal gene transfer is more important than previously conceived.

An ICE typically comprises three distinct modular structures that mediate its integration and excision, conjugation, and regulation. In addition, ICEs contain genes that confer specific phenotypes that are related to its existence in its hosts such as resistance to antibiotics and heavy metals. Therefore, ICEs are important vectors for the horizontal transfer of genetic information, thereby facilitating rapid bacterial evolution (Bi et al., 2011; Rodriguez-Blanco et al., 2012). The SXT/R391 family is the most extensively studied ICE family that was initially identified in *Vibrio cholerae* O139 (Waldor et al., 1996). To date, more than 40 members of this family have been identified in different clinical and environmental *Vibrio* species, as well as related gammaproteobacteria (Mata et al., 2011; Rodriguez-Blanco et al., 2012). The SXT/R391 family shares a core structure containing 52 conserved genes and a common chromosomal integration site at the 5′-end of the *prfC* gene, with other parts all integrating into 3′-ends of distinct element-specific tRNA gene loci (Wozniak et al., 2009; Wozniak and Waldor, 2010). All known elements contain variable DNAs that are inserted into specific positions of the backbone (hot spots) and encoding various beneficial traits such as antibiotic resistant determinants including but not limited to *floR, strBA, sul2, dfrA1, dfr18, tetA*′, *kanR*, and an emerging extended-spectrum cephalosporin resistance gene *bla*_CMY−2_ (Harada et al., 2010). SetR, a homolog of the phage λ repressor CI, derepresses the master transcriptional activators required for SXT transfer, including the *int* and *tra* operons (Daccord et al., 2012). Beaber et al. (2004) have shown that DNA-damaging agents mitomycin C and quinolinones which induce the host SOS response, increase the transfer of SXT by several hundred times. The horizontal dissemination of antibiotic resistance genes through ICEs under selective pressure poses new challenges to the use of antimicrobial agents. Extensive knowledge of SXT/R391 family ICEs prompted us to explore the genetic features of other family ICEs.

Comparative analysis of the genomes of different isolates of *Streptococcus agalactiae* has revealed that nearly 2/3 of its regions of diversity in the isolates consist of ICEs or ICE-like elements (Brochet et al., 2008), which include two of the largest families, namely, Tn916 and ICE*Sa*2603. The Tn916 family is extensively distributed across various organisms, including important human pathogens such as *Enterococcus faecalis, Clostridium difficile, Staphylococcus aureus*, and *Streptococcus* spp. (Roberts and Mullany, 2011). Furthermore, its transfer mechanism has been well studied (Roberts and Mullany, 2009). In contrast, the ICE*Sa*2603 family, although identified in various *Streptococcus* species, including *S. agalactiae* and *Streptococcus suis*, has not been comprehensively investigated. In the current study, we first comparatively analyzed 13 ICEs of the ICE*Sa*2603 family and two ICE*Sa*2603 family-like ICEs to perform an in-depth genetic characterization of the core genes that are present in all members of this ICE family. Next, we investigated the potential accessory functions encoded by the variable DNA harbored by these mobile elements as well as the conserved *att* site for integration and the *oriT* site for origination transfer. Phylogenetic analyses of the 30 core genes allowed us to classify the 15 ICEs were into four subgroups. Furthermore, the distribution of these ICEs was determined in *S. suis* and *S. agalactiae.* Finally, an experimental transfer of a swine origin 89K-like subgroup transferable ICE, ICE*Ssu*HB1011, was introduced. It is worth noting that isolates with *erm*(B)- and *tet*(O)-carrying transferable 89K-subtype ICEs were detected in different swine and bovine farms of China, thereby disseminating tetracycline and macrolide resistance genes and contributing to human pathogenesis (Tang et al., 2006).

## Materials and methods

### Bacterial strains and genomic DNA extraction

A total of 73 *S. suis* isolates from swine (including one *S. suis* strain from ATCC) and 21 *S. agalactiae* isolates from bovine (including one *S. agalactiae* strain from ATCC) were used in the present study (Table S1). Isolates were routinely grown overnight on tryptic soya broth or agar plates supplemented with 5% calf serum at 37°C. The bacterial culture was centrifuged (10,000 g for 5 min at room temperature), and the pellets were harvested and resuspended in TE buffer (10 mM Tris-HCl, 1 mM EDTA, pH 8.0) supplemented with lysozyme (5 mg/mL) and incubated for 30 min at 37°C. Genomic DNA was prepared using a Bacteria DNA kit (Omega, Norcross, GA, USA), following the manufacturer's instructions. The extracted DNA was used as template for PCR and sequencing.

### *In silico* identification of the ICE*Sa*2603 family

To identify and compare the ICE*Sa*2603 family ICEs, the reference genomes of ICE*Sa*2603 family were obtained from NCBI (Table 1). ICE*Sa*2603, ICE*Sde*3396, ICE*Sdy*12394-1, ICE*Spa*43144-1, ICE*Sth*JIM8232-1, ICE*Ssu*32457, ICE*Ssu*BM407-2, and ICE*Ssu*SC84 were previously classified as members of the ICE*Sa*2603 family (Bi et al., 2011; http://db-mml.sjtu.edu.cn/ICEberg/index.php). ICE*Sa*09mas018883, ICE*Ssu*05ZYH33-1 (also designated as 89K according to its size), ICE*Ssu*98HAH33-1, ICE*Ssu*D9, ICE*Ssu*SS12, and ICE*Ssu*HB1011 were grouped into this family because these encode the integrase gene that is closely related to *int*_ICE*Sa*2603_ and share significant sequence alignment and syntenic core structure. ICE*Ssu*T15 and ICE*Slu*van were identified as ICE*Sa*2603 family-like ICEs because these encoded a serine recombinase (SR) family integrase gene instead of the tyrosine family integrase gene, *int*_ICE*Sa*2603_.

**Table 1:** **Properties of the ICE*Sa*2603 family ICEs**.

**ICE name**	**Host strain**	**Accession**	**Size (bp)**	**% GC**	**Origin**	**References**
ICE*Sa*2603	*S. agalactiae* 2603V/R	AE009948.1	54349	38	United States	Tettelin et al., 2002
ICE*Sde*3396	*S. dysgalactiae* subsp. *equisimilis* NS3396	EU142041.1	63668	31	Australia, 2001	Davies et al., 2005, 2009
ICE*Sdy*12394-1	*S. dysgalactiae* subsp. *equisimilis* ATCC12394	CP002215.1	50568	39	United States, 1939	Suzuki et al., 2011
ICE*Spa*43144-1	*S. pasteurianus* ATCC43144	AP012054.1	58476	37	United States	Lin et al., 2011
ICE*Sth*JIM8232-1	*S. thermophilus* JIM8232	FR875178.1	50501	37	France, 2002	Delorme et al., 2011
ICE*Sa*09mas018883	*S. agalactiae* 09mas018883	HF952104.1	46903	37	–	Zubair et al., 2013
ICE*Ssu*32457	*S. suis* 32457	FR823304.2	54879	50.5	Italy, 2007	Palmieri et al., 2012
ICE*Ssu*05ZYH33-1	*S. suis* 05ZYH33	CP000407.1	88851	37	China, 2005	Chen et al., 2007
ICE*Ssu*98HAH33-1	*S. suis* 98HAH33	CP000408.1	89154	37	China, 1998	Chen et al., 2007
ICE*Ssu*SC84	*S. suis* SC84	FM252031.1	89165	37	China, 2005	Holden et al., 2009
ICE*Ssu*BM407-2^a^	*S. suis* BM407	FM252032.1	80320	36	Vietnam, 2004	Holden et al., 2009
ICE*Ssu*D9	*S. suis* D9	CP002641.1	55989	39.8	China	Zhang et al., 2011
ICE*Ssu*SS12	*S. suis* SS12	CP002640.1	64284	38.2	China	Zhang et al., 2011
ICE*Ssu*T15^a^^,^^b^	*S. suis* T15	CP006246.1	71412	36.3	–	–
ICE*Slu*van^b^	*S. lutetiensis* 5-F9	HE963029.1	94189	44	Netherlands	Bjorkeng et al., 2013
ICE*Ssu*HB1011^c^	*S. suis* HB1011	–	–	–	China, 2010	This study

a The SNF2 family protein was absent (ICESsuBM407-2) or partially absent (ICESsuT15).

b ICESsuT15 and ICESluvan with a serine recombinase (SR) family integrase were referred as ICESa2603 family-like ICEs.

c ICESsuHB1011 sequence was not complete.

### Phylogenetic analyses

**Figure 1:**
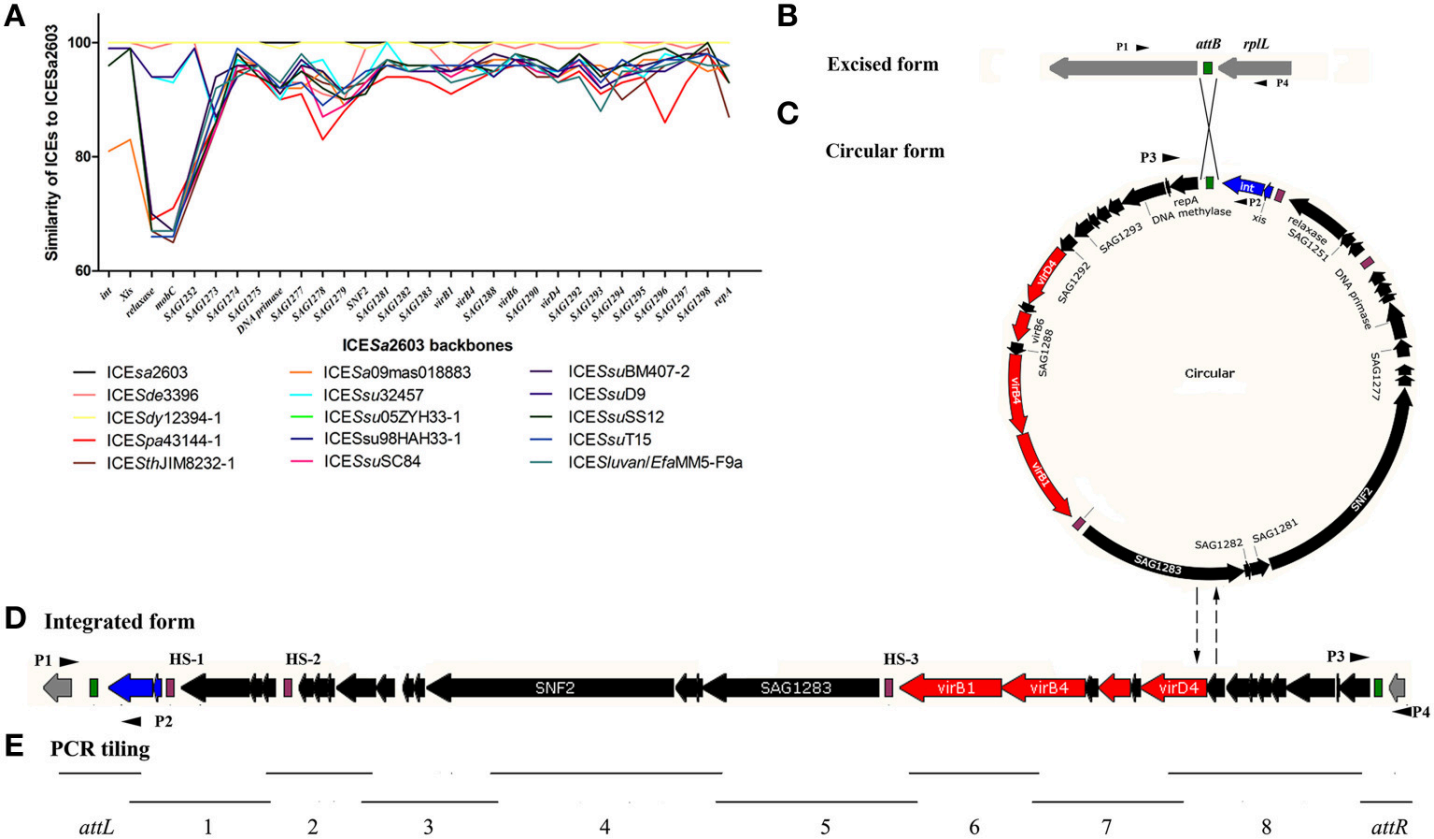
**Core genes identity (%) of the ICE*Sa*2603 family and schematic diagram of the integration and excision forms**. The *att*B **(B)**, *att*ICE **(C)**, *att*L, and *att*R **(D)** sites are shown as green rectangles. The core genes are shown by navy (*int* and *xis*), red (T4SS genes), and black (other core genes). The HS sites are shown as purple rectangles. **(A)**; Core genes identity (%) of each ICE of ICE*Sa*2603 family to that of ICE*Sa*2603. **(B–D)**; Schematic diagram of the integration and excision forms of the ICE*Sa*2603 family. **(E)**; Schematic diagram of PCR tiling to detect the presence of ICEs fragments.

The ICE*Sa*2603 family and family-like ICEs were aligned using ClustalW (Larkin et al., 2007) with default settings. Nucleotide and amino acid conservations were assessed using the appropriate BLAST algorithms. MAUVE 2.4 (Darling et al., 2004) and ACT software (Carver et al., 2005) were used to identify core genes. The core genes of each ICE were further aligned with the corresponding genes of ICE*Sa*2603 to calculate nucleic acid percentage identity (Figure 1A).

To obtain an overview of the relationship of the ICE*Sa*2603 family and family-like ICEs and its possible evolution, phylogenetic trees were generated from the alignment of whole core genes or each core gene using the neighbor-joining method with bootstrapping (*N* = 1000) parameters by thesoftware, MEGA6 (Tamura et al., 2013) and FigTree v1.4.2.

### Evaluation of the *att* and *oriT* sites

To identify the putative *att* sites, genomic sequences of the ICE-carrying strains were analyzed for the presence of directly repeated sequences flanking the ICEs. The ICE*Sa*2603 family-like ICE*Ssu*T15, where there are no conserved directly repeated sequences presented, the putative *att* site was predicted by comparing the junction sequence with *S. suis* P1/7.

The predicted *oriT* site of ICE*Ssu*05ZYH33-1 was first identified by Li et al. (2011). We determined that the *oriT* site is conserved in all other ICE*Sa*2603 family members. MEGA 6 (Tamura et al., 2013) and BioEdit v7 were used to align and mapthe *oriT* site the members of the ICE*Sa*2603 family.

### PCR and sequencing

To determine the presence of ICE*Sa*2603 family ICEs in *S. suis* and *S. agalactiae*, primers for the ICE*Sa*2603 core genes were designed and used in PCR analysis (Table S2). The integrated form and the extrachromosomal circular form of the ICEs of *S. suis* and *S. agalactiae* were detected by using combination primers, P1–P4 (Figures 1B–D and Table S2). PCR was also performed to detect the presence of ICE fragments in *S. suis* and *S. agalactiae* by PCR tiling assay as described previously (Sitkiewicz et al., 2011) using specific oligonucleotide primers (Figure 1E and Table S2). The products of a representative *S. suis* strain, HB1011, with the ICEs backbone as determined by PCR tiling were sequenced by Genewiz, Inc. (Suzhou, Jiangsu, China).

### Antimicrobials and susceptibility tests

Tetracycline, erythromycin, streptomycin, and rifampin (Sigma, St. Louis, MO, USA) were used in the present study. MICs were determined according to the guidelines described by the Clinical and Laboratory Standards Institute (CLSI, 2010).

### Conjugation experiments

*S. suis* BAA-853 and *S. agalactiae* G9, which were susceptible to tetracycline and erythromycin, were used to generate a rifampin- and streptomycin-resistant phenotype by using the stepwise-induced method with sub-MICs of rifampin and streptomycin (Haenni et al., 2010). In the mating experiments, the above two induced strains (tetracycline- and erythromycin-susceptible but rifampin- and streptomycin-resistant) were used as recipients and *S. suis* HB1011 (tetracycline- and erythromycin-resistant but rifampin- and streptomycin- susceptible) with the ICE*Ssu*HB1011 was utilized as donor. Filter mating assays were performed as previously described (Li et al., 2011). The transconjugant was further confirmed by PCR, sequencing, and MLST typing.

## Results

### General features of the ICE*Sa*2603 family

**Figure 2:**
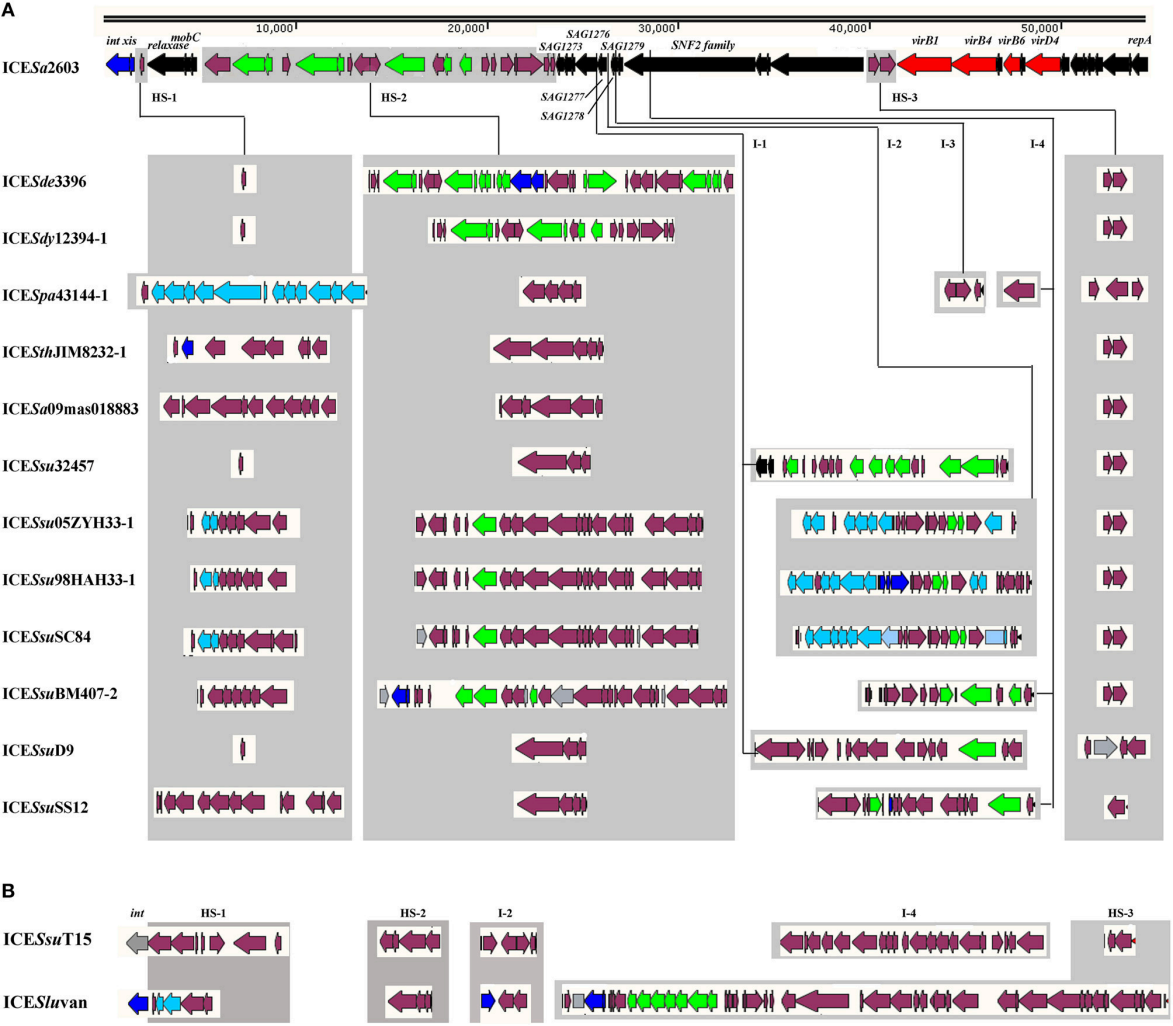
**Genomic organization of the ICE*Sa*2603 family**. HS-1, HS-2, and HS-3 were the hotspots of ICE*Sa*2603 family, which were presented in all ICEs. I-1, I-2, I-3, and I-4 could also receive additional genes in part of the ICEs. Genes are shown in different colors: black, ICEs core genes; navy, *int* and *xis*; azure, nisin operon genes; red, T4SS genes; green, antibiotic and heavy metal resistance genes; purple, other cargo genes. **(A)**; ICE*Sa*2603 family ICEs. **(B)**; ICE*Sa*2603 family-like ICEs.

A list of 13 ICEs of the ICE*Sa*2603 family and two ICE*Sa*2603 family-like ICEs from the NCBI complete genome database were analyzed and compared in this study (Table 1). An ICE that encodes an integrase gene closely related to *int*_ICE__*Sa*__2603_, defined as having >60% gene or protein homology, and has significant sequence alignment (60% nucleic acid identity of core genes) and syntenic core structure were classified as a member of ICE*Sa*2603 family (Bi et al., 2011; Figure 2A) (http://db-mml.sjtu.edu.cn/ICEberg/index.php). Although ICE*Ssu*T15 and ICE*Slu*van contained the backbone sequences of the ICE*Sa*2603 family, its integrase genes belonged to the SR family instead of the tyrosine family site-specific integrase of *int*_ICE__*Sa*__2603_, and these were referred to as ICE*Sa*2603 family-like ICEs (Figure 2B). The strains, which were originally isolated from different countries around the world, belonged to six species of the *Streptococcus* spp. (Table 1).

The ICEs of the ICE*Sa*2603 family were first compared by using the MAUVE 2.4 (Darling et al., 2004) and ACT software (Carver et al., 2005) to visualize conserved and variable regions. All ICEs, with sizes ranging from 46,903 bp (ICE*Sa*09mas018883) to 89,165 bp (ICE*Ssu*SC84), shared a common conserved structure and had variable regions (Table 1 and Figure 2A). The syntenic core structure of the ICE*Sa*2603 family backbone (33 kb) contained 30 conserved core genes (Figures 1A, 2). There were three sites within the conserved ICE*Sa*2603 family structure for inserting variable DNAs in all of the ICEs (Figure 2). The three sites were named as HS-1, HS-2, and HS-3 (i.e., hotspots) for ICE acquisition of new genes of resistance to antibiotics and heavy metals. The three hotspots were located in the intergenic region of the ICE backbone, suggesting that the acquisition of these variable DNA regions did not interrupted core ICE gene. Additional DNA was inserted into the 3′-end non-conserved region of gene *SAG1277* (Gene ID: 1014084), *SAG1278* (Gene ID: 1014085), *SAG1279* (Gene ID: 1014086), or *SNF2* (Gene ID: 1014087) of some ICEs (Figure 2). The four inserted sites were designated as I-1, I-2, I-3, and I-4, respectively. The function of SAG1277, SAG1278, and SAG1279 remains unclear.

### The ICE core genes

The present study has identified 30 core genes in 13 ICEs, which comprised the recombination module, the conjugation module, and various other genes of unknown functions. Integrases were members of the tyrosine recombinase family that contained a signature R·· H·· RH·· Y active site residue within its C-terminal catalytic domain. ICE*Sa*2603 family integrase Int_ICE__*Sa*__2603_ mediates site-specific recombination between identical *att*ICE and *att*B sites and integration into the 3′-end of 50S ribosomal subunit protein L7/L12 gene (*rplL*) (Haenni et al., 2010), which was determined to be conserved in all *Streptococcus* spp. (Table S3).

The conjugative transfer module of the ICE*Sa*2603 family was grouped into two parts clustered. The first cluster comprised 3 genes (*SAG1250, SAG1251, and SAG1252* in *S. agalactiae* 2603V/R), which were annotated as *Tn5252 orf4 relaxase, Tn5252 orf9 mobC*, and *Tn5252 orf10*, respectively. Protein SAG1250 showed similarities to various prokaryotic DNA relaxases that covalently bind to and nick the DNA at the origin of transfer (*oriT*) with the help of the auxiliary MobC to initiate DNA processing (Lanka and Wilkins, 1995), which include ORF4 relaxase (AAC98434.1) of conjugative transposon *Tn5252* (52% identity and 72% similarity) and MobA (AAQ55244.1) of *S. aureus* plasmid pC223 (22% identity and 41% similarity). Protein SAG1251, which is located adjacent to relaxase, shared 57% identity and 79% similarity to *Tn5252* ORF9 MobC (AAC98435.1) and 27% identity and 50% similarity to MobC (AAQ55243.1) of *S. aureus* plasmid pC223. The other part of the conjugation module belonged to a type IV secretion system (T4SS). The VirD4 coupling protein (40% identity to *Agrobacterium tumefaciens* VirD4), which functions as a substrate receptor, binds to the ICE DNA-relaxase complex (relaxosome) and presents it to the mating pair formation (Mpf) channels. Compared to *Agrobacterium tumefaciens* Vir T4SS (Christie, 1997), which are assembled from virB1–VirB11, only VirB1 (transglycosylase, 18% identity), VirB4 (ATPase, 41% identity), and VirB6 (inner-membrane protein, 12% identity) have been identified among the Mpf channels of the ICE*Sa*2603 family (Figure 2).

Additional conserved genes were located adjacent to the T4SS genes. Upstream of the *virD4* gene were genes coding for cytosine DNA methylase (*SAG1297*), replication initiation factors (*SAG1299*), and other unknown conserved genes, whereas downstream of the cluster were genes of the *SNF2* family (*SAG1280*), DNA primase (*SAG1276*), and other unknown conserved genes.

### Variable DNA regions

In addition to conserved core genes in all ICEs analyzed, variable DNA regions were also detected in the ICE backbone sequences (Figure 2). Most of the variable DNA sequences were inserted at three intergenic hotspots (HS), namely, HS-1, HS-2, and HS-3. The DNA content of various ICEs in HS was highly identical. For example, ICE*Sa*2603, ICE*Sde*3396, ICE*Sdy*12394-1, ICE*Ssu*32457, and ICE*Ssu*D9 had identical contents in HS-1. The DNA contents in HS-2 were more variable (only ICE*Ssu*32457 and ICE*Ssu*D9 shared identical genes), whereas in HS-3, these were more conserved (only ICE*Spa*43144-1, ICE*Ssu*D9, and ICE*Ssu*SS12 varied from the other ICEs). There were other exogenous DNA insertion sites (I), namely, I-1, I-2, I-3, and I-4 in at the 3′-end of the non-conserved region of genes *SAG1277, SAG1278, SAG1279*, and *SNF2* in some ICEs, respectively. For example, ICE*Ssu*32457 and ICE*Ssu*D9 had exogenous DNAs in I-1, whereas ICE*Ssu*05ZYH33-1, ICE*Ssu*98HAH33-1, ICE*Ssu*SC84, ICE*Ssu*T15, and ICE*Slu*van had variable DNAs in I-2. Additional DNA inserts were identified at the I-3 site in ICE*Spa*43144-1 and at the I-4 site in ICE*Spa*43144-1, ICE*Ssu*BM407-2, ICE*Ssu*SS12, and ICE*Ssu*T15.

Other notable accessory genes in this family were identified as resistance genes for antibiotics and heavy metals, intact or remnant lantibiotic biosynthesis operon, and other MGEs such as IS elements and Tn916-like elements (Figure 2). The antibiotic and heavy metal resistance genes in ICEs are presented in Table 2. ICE*Sa*2603, ICE*Sde*3396, and ICE*Sdy*12394-1 carry heavy metal resistance genes, whereas ICEs from *S. suis* mostly contain macrolide resistance gene *erm*(B) and/or tetracycline resistance gene *tet*(M) or *tet*(O). ICE*Spa*43144-1, ICE*Ssu*05ZYH33-1, ICE*Ssu*98HAH33-1, ICE*Ssu*SC84, and ICE*Slu*van had remnant nisin ORFs, in which a two-component signal transduction system nisK/nisR, is essential for full virulence of highly invasive *S. suis* serotype 2 (Li et al., 2008; Wang et al., 2014). In the first four ICEs, remnant nisin ORFs were presented in two variable regions at the insertion sites of HS-1 and I-2 (Figure 2A).

**Table 2:** **Attachment site analysis and resistance profile of the ICE*Sa*2603 family**.

**ICEs name**	**% Identity to *Int*_2603_**	**% Identity to *rplL*_2603_**	***att*B (3)**	**Putative *att* sites**	**Resistance profile**
ICE*Sa*2603	100	100	*rplL*	TTATTTAAGAGTAAC	Hg, Cd, Cu
ICE*Sde*3396	100	–	–	–	Cd, Cu, As
ICE*Sdy*12394-1	100	84	*rplL*	TTATTTAAGAGTGAT	Cd, Cu
ICE*Spa*43144-1	96	84	hypothetical protein	TTATTTAAGAGTAAC	
ICE*Sth*JIM8232-1	96	88	*rplL*	TTATTTAAGAGTAAC	
ICE*Sa*09mas018883	81	99	*rplL*	TTATTTAAGAGTAAC	
ICE*Ssu*32457	99	–^a^	*rplL*	TTATTTAAGAGTAAC	*erm*(B), *aad*E, *aph*A, *tet*(40), *tet*(O/W/32/O)
ICE*Ssu*05ZYH33-1	96	84	*rplL*	TTATTTAAGAGTAAC	*tet*(M), *aad*E
ICE*Ssu*98HAH33-1	96	84	*rplL*	TTATTTAAGAGTAAC	*tet*(M), *aad*E
ICE*Ssu*SC84	96	84	*rplL*	TTATTTAAGAGTAAC	*tet*(M), *aad*E
ICE*Ssu*BM407-2	96	84	*rplL*	TTATTTAAGAGTAAC	*tet*(M), *tet*(L), *tet*(O), *erm*(B), *aad*E, *cat*
ICE*Ssu*D9	99	84	*rplL*	TTATTTAAGAGTAAC	*tet*(O), *erm*(B)
ICE*Ssu*SS12	96	84	*rplL*	TTATTTAAGAGTAAC	*tet*(O), *erm*(B)
ICE*Ssu*T15^b^	–	84	*SSU0877* in *S. suis* P1/7	CCTCTTATGTCAAGTAACTG (*attL*) CATTATTATGACACAATCCC (*attR*)	
ICE*Slu*van^b^	–	–^a^	*rumA*	CACGTGGAGTGCGTAGTGTT (*attL*) TTCTCAAGGACCAGACAACA (*attR*)	*van* resistance operon
ICE*Ssu*HB1011^c^	98	–	*rplL*	TTATTTAAGAGTAAC	*tet*(O), *erm*(B)

a The rplL sequences were not indicated.

b The ICESsuT15 and ICESluvan's Int belonging to serine recombinase (SR) family shared low homology with the ICESa2603 tyrosine family site-specific integrase. ICESluvan was inserted in rum, and the att site was experimentally confirmed by Bjorkeng et al. (2013). ICESsuT15 was inserted in the 344 sites of SSU0877 in S. suis P1/7, the putative att site was predicted, and the Int of ICESsuT15 and ICESluvan may recognize CA dinucleotide in certain region of chromosome sites.

c ICESsuHB1011 sequence was not complete and the rplL sequences were not known.

MGEs were also found to be inserted in HS or I sites. For example, IS116 and IS4 family IS elements were inserted in the I-4 region of ICE*Spa*43144-1 and ICE*Ssu*D9, respectively (Figure 2A). Moreover, a Tn916-like element was inserted in HS-2 of ICE*Ssu*05ZYH33-1, ICE*Ssu*98HAH33-1, ICE*Ssu*SC84, and ICE*Ssu*BM407-2 (Figure 2A). A Tn1549-like element was inserted in I-4 of ICE*Slu*van, thereby forming a mosaic ICE ((Bjorkeng et al., 2013); Figure 2B). The integrase of ICE*Slu*van, which belongs to theSR family instead of the tyrosine recombinase family of Int_ICE__*Sa*__2603_, resulted in the alteration of the insertion site, thus generating a new ICE (ICE*Sa*2603 family-like ICE).

### The *att* site and *oriT* site

**Figure 3:**
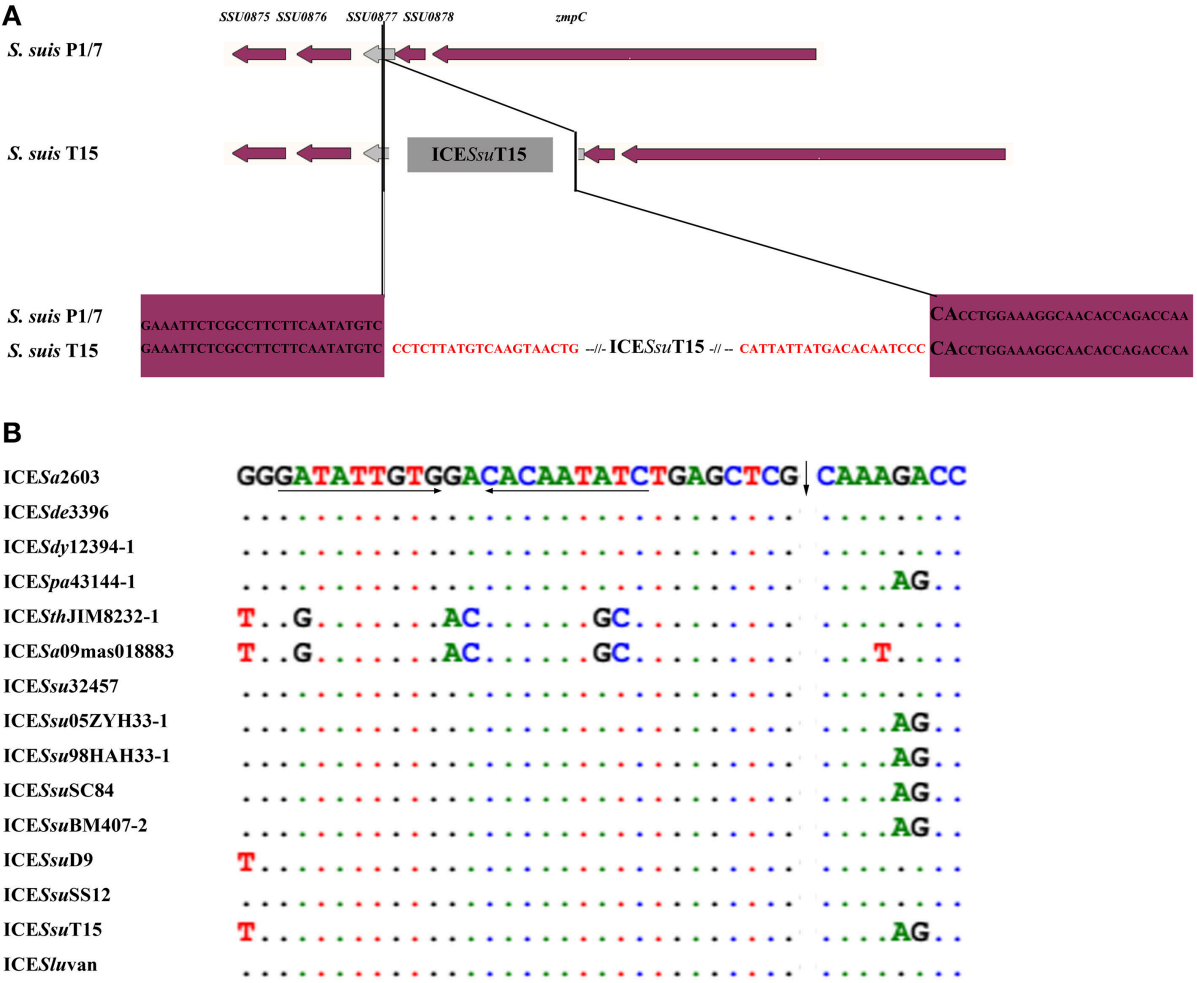
***att* site of ICE*Ssu*T15 (A) and *oriT* site of the ICE*Sa*2603 family (B)**. **(A)**; ORFs in purple represent the chromosome genes while in gray shows the integraing site of ICE*Ssu*T15, the putative *att* sequence was indicated in red. **(B)**; the *oriT* site of the ICE*Sa*2603 were listed in the top, the bases different from ICE*Sa*2603 were indicated. Arrows under the sequences represent the locations of inverted repeats, and vertical arrows show the nick sites.

ICEs of the ICE*Sa*2603 family were found to specially integrate at the 3′-end of the *rplL* gene of *Streptococcus* spp., by recognizing the *att* sequence, TTATTTAAGAGTAAC (Table 2). Although different identities of *rplL* gene were observed, the *att* sequence (15 bp from the 3′-end of the *rplL* gene) appeared more conserved in *Streptococcus* spp. (Table 2 and Table S3), thus providing the sequence basis for its distribution among members of the family. ICE*Ssu*T15 and ICE*Slu*van with Int and belonging to SR instead of tyrosine family integrase of ICE*Sa*2603 family were assumed to have a different *att* site. As shown in Figure 3A, there were no consensus insertion sequences have been identified in ICE*Ssu*T15.

A ~200 bp highly conserved region (92–100% identity) highlighted in the visual comparison with Mauve 2.4 (Darling et al., 2004) is considered to be the putative the *oriT* region. The left adjacent region was *SAG1252* (*Tn5252 orf10*, 65–100% identity) gene that showed relatively low identity and the right adjacent region was a variable DNA hotspot site. In all ICEs except for ICE*Sth*JIM8232-1 and ICE*Sa*09mas018883, the *oriT* site contained a 9-nt inverted repeat sequence, GATATTGTG, which served as a binding site for relaxase, and the precise nickase cleavage site was predicted to reside at position 5′-CTCG/CAAA (Figure 3B).

### Phylogenetic analyses of the ICE core genomes

To analyze the evolution of the ICE core genomes, we first compared the identity of each ICE core genes to the corresponding ICE*Sa*2603 genes (Figure 1A). Most ICE core genes exhibited a >90% identity at the nucleotide level. On the other hand, the less conserved genes were located at the junction between core genes and variable region. For example, the DNA processing genes (*SAG1250*–*SAG1252*) of the conjugative transfer module between HS-1 and HS-2 were much less conserved (65–72%) than other core genes, whereas the *oriT* site remained highly conserved.

**Figure 4:**
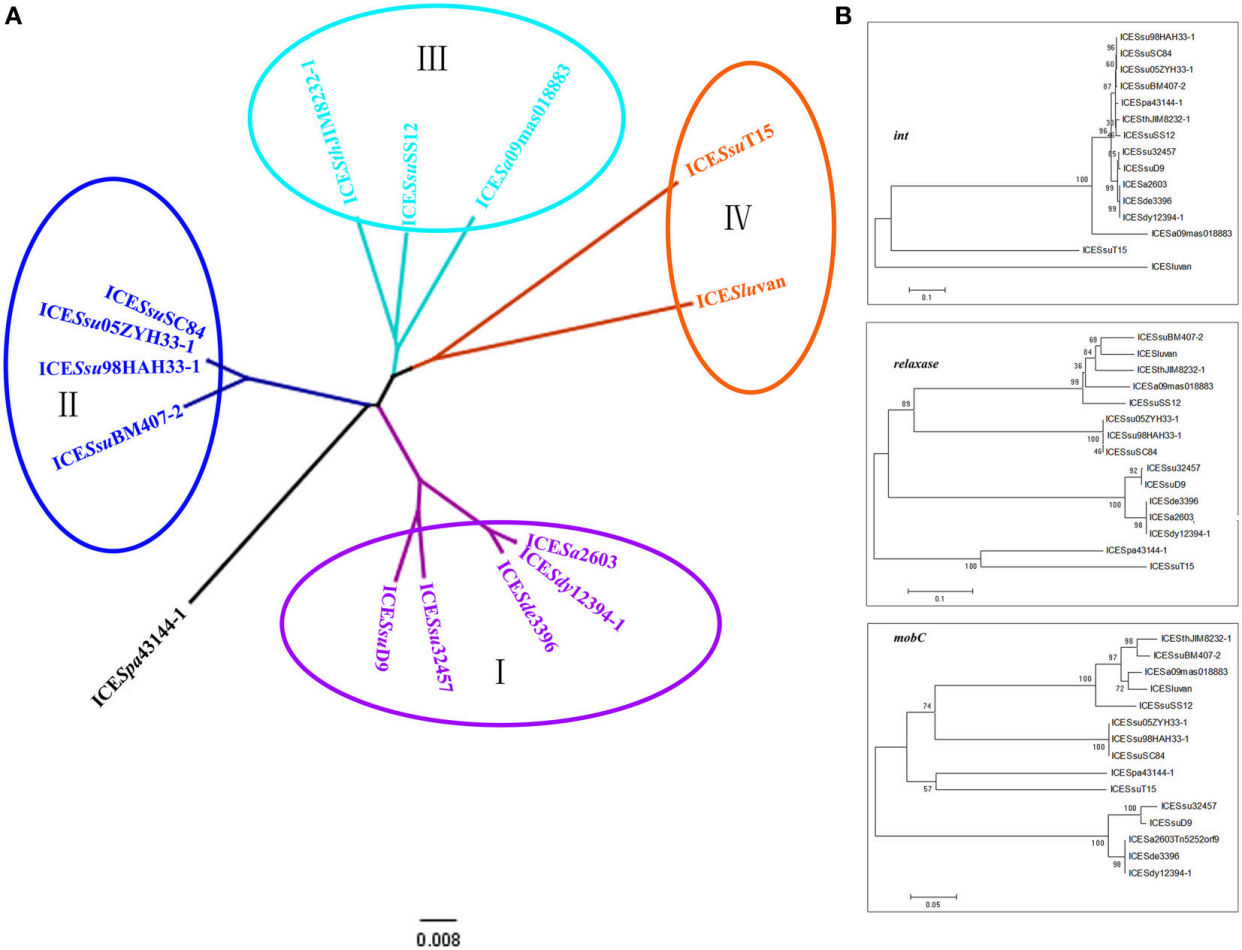
**Phylogenetic tree of ICE*Sa*2603 family. (A)**, phylogenetic tree generated from 30 core genes. Four subgroups (I–IV) can be classified; **(B)**, phylogenetic tree of *int, relaxase*, and *mobC*.

Next, we generated phylogenetic trees based on all the 30 core genes (Figure 4A) or individual representative genes (Figure 4B). The ICEs were then further divided into four subgroups: ICE*Sa*2603 family groups I–III (ICE*Sa*2603-like, 89K-like, and ICE*Sth*JIM8232-1-like) and ICE*Sa*2603 family-like group IV. Interestingly, these subgroups also showed variable features: The ICEs of group IV could be distinguished from that of other groups based on the *int*, only one regulatory gene (*SAG1249*) was found in HS-1 region of group I ICEs (ICE*Sa*2603-like); and a transposon Tn916 was inserted into HS-2 region of group II ICEs, thereby generating mosaic ICEs (Figures 2, 4A).

To gain more insights into the evolution of individual core genes, we created phylogenetic trees for each core gene. However, most core genes were determined to be highly conserved, thus making it difficult to generate a reliable tree. Hence, we concentrated on the less conserved genes and selected *int, relaxase*, and *mobC* for phylogenetic analyses. Figure 4B shows the two subgroups of *int*: the tyrosine recombinase family *int* genes, which were highly conserved and present in all ICE*Sa*2603 families (88–100% identity), and the SR family *int* genes that were present in ICE*Sa*2603 family-like ICEs. According to the tree of *relaxase* and *mobC*, all ICEs in group I were aligned together and shared ~94% similarity, whereas other ICEs generated distinct branching patterns (with < 80% similarity).

### Distribution of members of the ICE*Sa*2603 family in *S. suis* and *S. agalactiae*

To evaluate the presence of ICEs in *S.* s*uis* isolates from pigs and *S. agalactiae* isolates from cows in China, several core ICEs genes were detected (Table 3 and Table S1). Of the 73 *S. suis* isolates screened, 31 were determined to contain ICEs based on the presence of T4SS genes (*virB1, virB4, virB6*, and *virD4*), in which 80.5% (25/31) also carried *int*_ICE__*Sa*__2603_. Similarly, 38.1% of the tested *S. agalactiae* isolates harbored T4SS genes, amongst which 62.5% (5/8) were *int*_ICE__*Sa*__2603_-positive. Different primer pairs P1–P4 were employed and identified the integration/excision form of ICE*Sa*2603 family in the present study (Table 3). Table 3 shows that 88% (22/25) of the ICE*Sa*2603 family strains were excised from the chromosomes, thereby forming circular intermediates prior to its conjugative transfer into recipient cells.

**Table 3:** **Distribution of ICE*Sa*2603 family core genes in *S. suis* and *S. agalactiae***.

**Strains**	***int*_ICE_*_*Sa*_*_2603_**	***DNA primase***	**T4SS genes**				***repA***	**ICE at *rplL* site confirmed by PCR^a^**
			***virB1***	***virB4***	***virB6***	***virD4***		
*S. suis* (*n* = 73)^b^	25	65	32	32	32	31	30	22
*S. agalactiae* (*n* = 21)^c^	5	19	8	8	8	8	8	5

a The extra chromosomal form and integrating form of ICEs can be seen in Figures 1B–D.

b S. suis were isolated from swine in china (Table S1).

c S. agalactiae were isolated from bovine in china (Table S1).

To determine whether the ICEs were intact, PCR tiling was performed. Ten overlapping fragments were amplified using the long-PCR method (Figure 1E and Table S2). Table 4 and Table S1 showed that almost all fragments of ICE core regions (fragments 4, 6, 7, and 8) could be amplified in macrolide- and tetracycline-resistant *S. suis*, whereas other fragments (1, 2, 3, 5) linking the variable regions (HS-1–HS-3 and I-1–I-4) were more variable. PCR tiling also detected various ICE subgroups. For example, the strain with fragments 2–8, but without fragments *attL*, 1, and *attR*, belonged to subgroup IV (ICE*Sa*2603 family-like); Furthermore, subgroup I could be distinguished from other subgroups based on the size of fragment 1.

**Table 4:** **Analyses of ICE*Sa*2603-family fragments in part of macrolide and tetracycline resistant *S. suis* by PCR tiling assay**.

**Fragments^a^**	***attL***	**1**	**2**	**3^b^**	**4**	**5**	**6**	**7**	**8**	***att*R**
HB1004	+	+	-	-	+	+	+	+	+	+
HB1006	-	-	-	-	+	+	+	+	+	+
HB1011^c^	+	+	+	-	+	+	+	+^b^	+	+
HB1012	+	+	-	-	+	+	+	+	+	+
HB1013	-	-	-	-	+	+	+	-	-	-
NJ4	+	-	-	-	+	+	+	+	-	-
JS07002	+	+	-	-	+	-	+	+	+	+
JS07015	+	+	-	-	+	+	+	+	+	+
SC05017	-	-	-	-	+	+	+	+	+	-
SS2-XY	+	-	-	-	+	+	+	+	+	+
YY060816	-	-	-	-	+	+	+	+	+	-

a The PCR tiling assay and fragments can be seen in Figure 1E.

b Failure of amplifying this fragment, probably due to too long additional DNA segments inserted in the 4 regions (I-1 to I-4)

c The fragment 7 in HB1011 was ~2 kb larger than ICESa2603 core region.

### ICE*Ssu*HB1011, a swine origin 89K-like subgroup transferable ICEs

**Figure 5:**
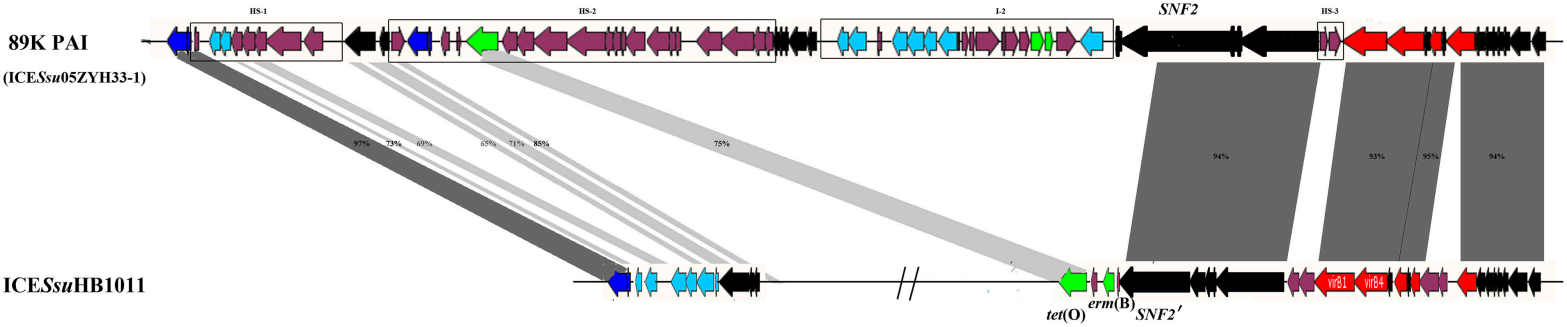
**ORF map of ICE*Ssu*HB1011 to the 89K PAI (ICE*Ssu*05ZYH33-1) of *S. suis* 05ZYH33**. 89K PAI (ICE*Ssu*05ZYH33-1) was proved to be a pathogenicity island response for the human *S. suis* Outbreak in Sichuan, China in 2005. Genes are shown in different colors: black, ICEs core genes; navy, *int* and *xis*; azure, nisin operon genes; red, T4SS genes; green, antibiotic and heavy metal resistance genes.

Because ICEs play an important role in the horizontal transmission of antibiotic resistance, we selected a swine-derived clinical macrolide- and tetracycline-resistant strain HB1011 (ST303, a new ST type) for subsequent conjugation experiments. The transfer of macrolide and tetracycline resistance from HB1011 to *S.suis* BAA-853 (ST1) was confirmed by sequencing of the PCR products (Figures S1, S2) and MLST typing. The MICs of erythromycin and tetracycline and PCR products of *erm*(B) and *tet*(O) in conjugant JH-1 confirmed that erythromycin and tetracycline resistance genes *erm*(B) and *tet*(O) were indeed transferred (Table S4). We further sequenced the tiling fragments of ICE*Ssu*HB1011, which is an 89K-like ICE (Figure 5). The results showed that *erm*(B) and *tet*(O) genes were inserted into the SNF2 gene (I-4, Figure 1).

## Discussion

ICEs are self-transmissible MGEs that encode the machinery for conjugation, as well as intricate regulatory systems to control excision from chromosomes and its conjugative transfer (Wozniak and Waldor, 2010). ICEs can be considered as mosaic elements that present the combined features of plasmids and bacteriophages. Similar to plasmids, ICEs transfer via conjugation and in parallel to phages, ICEs integrate into and replicate with its host chromosome (Burrus and Waldor, 2004). ICEs often carry accessory genes that encode for antibiotic resistance, virulence factors and various other functions. These are therefore important vectors for the horizontal dissemination of genetic information, thereby facilitating rapid bacterial evolution.

ICE*Sa*2603 is a ~54 kb ICE that was first discovered in *S. agalactiae* 2603V/R (Tettelin et al., 2002). It has the capacity to retain its features of transferability to *S. agalactiae, S. dysgalactiae* subsp *dysgalactiae*, and *S. uberis* (Haenni et al., 2010). Subsequently, ICE*Sa*2603-like ICEs have been detected in clinical isolates of *Streptococcus* spp. from both human and animal species from Europe and Asia (Table 1). Therefore, it is likely that the members of the ICESa2603 family have been distributed and have the ability to undergo horizontal transmission in *Streptococcus* spp. The number of ICESa2603 family ICEs and its host range may therefore further expand as additional research studies are currently being conducted. However, comparative genomics of ICE*Sa*2603 family ICEs and its evolution have not been reported. Here, we first comparatively analyzed 13 ICEs of the ICE*Sa*2603 family. The results showed that the ICE*Sa*2603 family has a backbone of 30 core genes that encode the core functions of integration/excision, conjugation and regulation.

The integration/excision modules of ICE*Sa*2603 consisted of Int_ICE__*Sa*__2603_ (SAG1247) and Xis_ICE__*Sa*__2603_ (SAG1248). Int_ICE__*Sa*__2603_ belongs to the tyrosine based site-specific recombinases. This family of integrase originated from bacterial phages and conjugate transposons. One member is the integrase from *Bacillus subtilis* conjugative transposon ICE*Bs*1, which mediates integration into the 3′-ends of tRNA-Leu loci (Lee et al., 2007). Int_ICE__*Sa*__2603_ shares 23% identity with Int_ICE__*Bs*__1_, but mediate site-specific recombination between identical *att*ICE and *att*B sites and the integration into the 3′-end of the 50S ribosomal subunit protein L7/L12 gene (Haenni et al., 2010). The *attB* sites (TTATTTAAGAGTAAC) of ICE*Sa*2603 family is conserved in *Streptococcus* spp. and even *Enterococcus faecium* and *Enterococcus faecalis*, which provided the sequence basis for the horizontal transfer of this family. To date, members of the ICE*Sa*2603 family has been identified in six species of *Streptococcus* spp. (Table 2). The ICE*Sa*2603 family-like ICEs, share 28 of 30 core genes with ICE*Sa*2603 family ICEs, which contain the SR family integrase, although a low level of identity was observed between ICE*Slu*van and ICE*Ssu*T15 (Figures 1A, 4B). A study on another SR Int_*Tn*5397_ has shown that the insertion site contains a central GA dinucleotide; however, no other consensus insertion sequences have been identified (Wang and Mullany, 2000). Based on the experimental transfer of ICE*Slu*van (Bjorkeng et al., 2013) and the analysis of ICE*Ssu*T15 (Figure 3A), we believe that *Int* of ICE*Slu*van and ICE*Ssu*T15 recognize the CA dinucleotide in certain regions of chromosomal sites. The ICE*Slu*van is capable of transmission, which provides an illustration that acquisition of a new integration/excision module (most likely via recombination) generates new types of ICEs (here we termed as ICE*Sa*2603 family-like ICEs). Xis, the adjacent recombination directionality factor (RDF), both facilitates excision and inhibits integration. It binds to the ends of the integrated element adjacent to Int binding sites. Deletion of the *xis* gene of ICE*Ssu*05ZYH33-1 demonstrated that xis stimulates, but is not essential for ICE*Ssu*05ZYH33-1 excision from chromosomes (Li et al., 2011).

The conjugation module of ICE*Sa*2603 family is similar with that of plasmids. When ICE*Sa*2603 transfers, a relaxase (SAG1250) first binds to and excises circular ICE DNA at *ori*T sites (Li et al., 2011). Thereafter, with the help of a coupling protein, MobC (SAG1251), the relaxase-DNA complex is presented to type IV coupling protein (T4CP) and the Mpf complex. Finally, the T4CPMpf complex transfers the ICE DNA to the receipt cell. The T4SS complex includes genes encoding for proteins similar to *A. tumefaciens* VirB1, VirB4, VirB6, and VirD4. Zhang et al. reported that these T4SS gene clusters were conserved both in composition and order in the genomes of various *Streptococcal* species and thus collectively described these as a Type–IVC secretion system (Zhang et al., 2012). Knockout of two key components genes (i.e., *virD4* and *virB4*) of the Type–IVC secretion system not only abolished its transferability but also eliminated the lethality of the highly virulent strain and impaired its ability to trigger a host immune response in infected mouse models (Li et al., 2011; Zhao et al., 2011). These results suggest that T4SS has retained its ancestral function as a conjugation module while its capacity to translocate effector protein(s) to the cell surface or into eukaryotic target cells undergoes evolution.

Our understanding of the mechanism underlying the regulation module of the ICE*Sa*2603 family is limited. Comparative analysis of the 30 core genes of ICE*Sa*2603 family ICEs has shown that no common regulators were conserved in all ICEs of this family. Instead, a gene (*SAG1249*) encoding a Cro/CI family transcriptional regulator was detected in all experimentally proven transferable ICEs, despite not being classified as core genes in the present study. This regulator possibly regulates ICE gene expression and ICE transfer (Beaber and Waldor, 2004). Recently, Xu et al. described a two-component system, NisK/NisR, in subgroup II (89K-subgroup, Figure 4A) ICEs that contributes to the virulence of *S. suis* serotype 2 (Xu et al., 2014). It is tempting to know whether this two-component system is involved in the regulation of transfer. A bacteriophage abortive infection (Abi) system, AbiE, which assists bacteria from being killed by bacteriophages, has been recently identified as a type IV toxin-antitoxin system in ICE*Sa*2603 (*SAG1284* and *SAG1285*) (Chopin et al., 2005; Dy et al., 2014). Dy et al. (2014) demonstrated that the AbiE system enables plasmid maintenance when these were introduced into pUC19. It is possible that the *abiE* operon and native promoter enable the maintenance of the circular form of ICE*Sa*2603, thereby increasing the frequency for ICE transfer.

Phylogenetic and comparative analyses have shown that most of the core genes of the ICE*Sa*2603 family exhibited 91–100% identity at the nucleotide level (Figure 1A). No detectable difference in the degree of conservation of most core genes was observed, which suggests equal selective pressures on core genes of the ICEs. However, some genes (i.e., *Int, xis, relaxase, mobC*, and *SAG1278*) exhibited lower degrees of conservation. The difference in the identity of the *Int* and *xis* genes, as earlier described, was probably generated by recombination. The *relaxase* and *mobC* genes can be divided into four subgroups (Figure 4B). The *relaxase* and *mobC* genes exhibited a high level of identity within subgroups (>80%), but a low identity between subgroups (65–80%). These results suggest that the different subgroups of *relaxase* and *mobC* genes (DNA processing module) might have also been generated by recombination of the entire DNA processing module. The relative low level of conservation of *SAG1278*, in which of the 3′-end is often the insertion site (I-2), might have been ascribed to the insertion of accessory genes, as indicated by the lower level of identity of this gene in each ICE. These results also suggest that individual core genes are exposed to different evolutionary pressures.

BLAST analysis of the core conjugation and integration module proteins of the ICE*Sa*2603 family showed that ICE*Sa*2603 shared some similarity with the *Streptococcus pneumoniae* conjugative transposon Tn5252 (Alarcon-Chaidez et al., 1997). Based on limited knowledge, we propose that ICEs were disseminated across a diverse range of hosts, thereby resulting in exposure to different selective stress. To survive stress, cargo genes encoding antibiotic resistance, nitrogen fixation, virulence factors, and various other functions were introduced into the HS and I sites. In the case of the ICE*Sa*2603 family, insertion of variable DNAs might have disrupted the core regulatory module gene (*SAG1249*), but another regulatory module gene could be functioned to compensate and restore stability in the chromosome (Li et al., 2008, 2011). Another benefit is that recombination of the core module increases the adaptability and diversity of ICEs or MGEs. Examples include the ICE*Sa*2603 family-like ICEs, ICE*Slu*van, in which only the conjugation module *int*_*Tyr*_–*xis* was replaced by SR family *int*_*SR*_ yet retained its transferability (Bjorkeng et al., 2013). A very recent study (Marini et al., 2015) has shown that mating between *S. suis* 32457 (donor) and *S. agalactiae* 2603V/R (recipient) yields a hybrid ICE, ICESa2603/ICESsu32457, and interestingly, could be transferred across various *Streptococcus pyogenes* strains. These findings strongly support the concept that recombination plays an important role in horizontal gene transfer and generates novel ICEs or even new types of MGEs. These findings and the above mentioned recombination/insertion/hybrid events further broaden range of ICE diversity. The mosaic patterns of the ICE*Sa*2603 family consisted of genes from groups A, B, and G *Streptococcus* organisms, *Faecalibacterium*, enterococci, and possibly *Listeria innocua* and *Enterococci faecalis*, indicating that recombination and mobilization events are key factors in the assembly of these families. The exchange of integration modules among ICEs or between ICEs and other integrating MGEs (e.g., phages) could potentially create new ICEs with altered insertion site specificities or modified host ranges.

Figure 4A shows that all four subgroups of the ICE*Sa*2603 family were detected in *S. suis*, which is indicative of its important role in the transmission of these types of ICEs of *S. suis*. Screening of ICE*Sa*2603 core genes showed that 42.5% of swine-derived *S. suis* isolates contained ICE T4SS genes and 80.5% of these belong to ICE*Sa*2603 family. These results suggest that *S. suis* isolates harbor ICE*Sa*2603 family ICEs that are extensively distributed in swine in China, which has become a challenge to the hog industry and even a threat to human health.

89K-like subgroup ICEs (ICE*Ssu*98HAH33-1 and ICE*Ssu*05ZYH33-1) were detected in two *S. suis* isolates and have caused two extensive human outbreaks of streptococcal toxic shock–like syndrome (STSLS) in China in 1998 and 2005 (Tang et al., 2006). Further studies (Li et al., 2008; Zhao et al., 2011; Zhong et al., 2014) have demonstrated that 89K is a pathogenicity island (PAI). Li et al. (2008) earlier showed that a two-component signal transduction system (SalK/SalR) is essential for full virulence of highly invasive *S. suis* serotype 2. SalK/SalR, which is also the regulatory module of the nisin lantibiotic operon, was inserted into the HS-1, and possibly acts as a regulator, together with SAG1249. Furthermore, knockout of three key components (*virB1, virB4*, and *virD4*) of T4SS significantly reduces but does not abolish the virulence of *S. suis* in a mouse model (Zhao et al., 2011; Zhong et al., 2014). These findings indicate that T4SS harbored by 89K PAI contributes to the development of STSLS and mediates the horizontal transfer of 89K. However, those types of ICEs did not contain genes that encode resistance to macrolides and tetracycline. In the present study, we observed that almost all of the macrolide- and tetracycline-resistant *S. suis* also contained T4SS genes, indicating that ICEs play an important role in spreading macrolide and tetracycline resistance in *S. suis*. The isolated strain HB1011, which was determined to harbor ICE*Ssu*HB1011 that carried *erm*(B) and *tet*(O), which was indicative of its multiple functions, including horizontal transfer resistance and pathogenicity. In addition, other strains of *S. agalactiae* and *S. dysgalactiae subsp. equisimilis* whose ICEs harbored T4SS that was similar to 89K did not cause STSLS. Therefore, it is of great importance to identify factors and mechanisms by which the T4SS of 89K mediates pathogenicity. It has been proposed that ICE*Sa*2603 family T4SS might have retained its ancestral function as a mobile DNA element, but have also evolved its capacity to translocate effector protein(s) to the cell surface or into eukaryotic target cells (Bhatty et al., 2013).

## Conclusions

In summary, the structures of the ICE*Sa*2603 family ICEs in isolates of different *streptococcal* species are clearly illustrated in this study by comparative analyzing 13 ICEs of the ICE*Sa*2603 family and two ICE*Sa*2603 family-like ICEs. Screening of ICE*Sa*2603 core genes shows that ICE*Sa*2603 family ICEs are distributed widely in *S. suis* and *S. agalactiae* in China and mainly belong to the 89K-subtype family ICEs. Furthermore, the spread of the ICEs containing resistance genes *erm*(B) and *tet*(O) among *S. suis* strains by conjugation had a profound effect on the horizontal transmission of antibiotic resistance to macrolides and tetracyclines. Further research is needed to characterize the mechanisms involved in triggering ICEs transfer intra- and inter-species.

## Author contributions

JH, DG, and LW developed the concept and designed experiments. JH, YL, KS, and LG performed the experiments and collected the data. JH, YL, and JK conducted all bioinformatics analyses. JH and LW prepared the manuscript. All authors have contributed to, seen and approved the manuscript.

### Conflict of interest statement

The authors declare that the research was conducted in the absence of any commercial or financial relationships that could be construed as a potential conflict of interest.
