# Supplementary material for: Comparative Genomic Analysis of the ICESa2603 Family ICEs and Spread of erm(B)- and tet(O)-Carrying Transferable 89K-Subtype ICEs in Swine and Bovine Isolates in China
Source: Front Microbiol. 2016 Feb 2;7:55. doi: 10.3389/fmicb.2016.00055 (PMC4735348; doi:10.3389/fmicb.2016.00055)
Supplement: Supplementary file 2 [file DataSheet2.docx]

Table S2 Primers used in this study

| Primers | Sequence (5’-3’) |  |
| --- | --- | --- |
| Primers used for PCR tiling assay | | |
| *int*-R | TGGTCTTTACGATACCAAT | ([Haenni et al., 2010](#_ENREF_1)) |
| oriT-F | CGGRGCCACCAAATTATCT | This stduy |
| *erm*B*-*F | ACTCGTGTCACTTTAATTCACC | This stduy |
| *erm*B*-*R | AATTGTTTACTTTGGCGTGTT | This stduy |
| *tet*O*-*F | ACGGAAAGTTTATTGTATACCAG | This stduy |
| *tet*O*-*R | CACAATACCTTGGAGCATC | This stduy |
| *SNF2-*F | TACTTCACTTTGAGACAGATG | ([Palmieri et al., 2012](#_ENREF_2)) |
| *SNF2-*R | TAGTTGAGTTACCGAGGC | ([Palmieri et al., 2012](#_ENREF_2)) |
| *virB-*F | GGTCGAATGGGTCTTTGTCT | ([Palmieri et al., 2012](#_ENREF_2)) |
| *virB-*R | GCTTGGTGTGTTGTGGGATC | ([Palmieri et al., 2012](#_ENREF_2)) |
| *repA-*F | GTGTCATCTCGTGGCTATTT | ([Palmieri et al., 2012](#_ENREF_2)) |
| Detecting integration/excision form of ICEs in *S. suis* | |  |
| P1(*hdy-*R) | GCGGTCGATAGGAACAACC | This stduy |
| P2(*int-*F) | AACAAAGACTCCAGCAGGTGA | This stduy |
| P3(*repA-*R) | GCCCCATCCTCATCAATCC | This stduy |
| P4(*rplL-*F) | AAAGTTGGCGTTATCAAAG | This stduy |
| Detecting integration/excision form of ICEs in *S. agalactiae* | |  |
| P1(*SAG1246-*R) | TCTCAACAAGTTCATGCAG | This stduy |
| P2(*int-*F) | AAATCAACCATTTGTCGCATC | This stduy |
| P3(*repA-*R) | TTTTATTTGCCATTCGACCTC | This stduy |
| P4(*rplL-*F) | GCTAAAGACTCATTTGACGTTG | This stduy |

Haenni, M., Saras, E., Bertin, S., Leblond, P., Madec, J.Y., and Payot, S. (2010). Diversity and mobility of integrative and conjugative elements in bovine isolates of Streptococcus agalactiae, S. dysgalactiae subsp. dysgalactiae, and S. uberis. *Appl Environ Microbiol* 76**,** 7957-7965.

Palmieri, C., Magi, G., Mingoia, M., Bagnarelli, P., Ripa, S., Varaldo, P.E., and Facinelli, B. (2012). Characterization of a Streptococcus suis tet(O/W/32/O)-carrying element transferable to major streptococcal pathogens. *Antimicrob Agents Chemother* 56**,** 4697-4702.

| species | 3’ of *rplL(attB* sites*)* | *rplL* genes identity |
| --- | --- | --- |
| *Streptococcus_suis*_05ZYH33_(CP000407) | TTATTTAAGAGTAAC | 100% |
| *Streptococcus_mitis*_B6_(NC_013853.1) | TTATTTAAGAGTAAC | 94% |
| *Streptococcus_pseudopneumoniae*_IS7493_(NC_015875.1) | TTATTTAAGAGTAAC | 94% |
| *Streptococcus_pneumoniae*_ATCC700669_(NC_011900.1) | TTATTTAAGAGTAAC | 93% |
| *Streptococcus_oligofermentans*_AS1.3089_(NC_021175.1) | TTATTTAAGAGTAAC | 92% |
| *Streptococcus_oralis*_Uo5_(NC_015291.1) | TTATTTAAGAGTAAC | 92% |
| *Streptococcus_parasanguinis*_ATCC15912_(NC_015678.1) | TTATTTAAGAGTAAC | 91% |
| *Streptococcus_gallolyticus_subsp._gallolyticus*_ATCC43143(NC_017576.1) | TTATTTAAGAGTAAC | 89% |
| *Streptococcus_gallolyticus_*UCN34(NC_013798.1) | TTATTTAAGAGTAAC | 89% |
| *Streptococcus_infantarius_subsp._infantarius_*CJ18_(NC_016826.1) | TTATTTAAGAGTAAC | 89% |
| *Streptococcus_salivarius_*JIM8777_(NC_017595.1) | TTATTTAAGAGTAAC | 89% |
| *Streptococcus_sanguinis_*SK36_(NC_009009.1) | TTATTTAAGAGTAAC | 89% |
| *Streptococcus_anginosus_*C238_(NC_022239.1) | TTATTTAAGAGTAAC | 88% |
| *Streptococcus_thermophilus_*JIM8232_(FR875178) | TTATTTAAGAGTAAC | 88% |
| *Streptococcus_intermedius_*B196_(NC_022246.1) | TTATTTAAGAGTAAC | 87% |
| *Streptococcus_macedonicus*_ACA-DC198_(NC_016749.1) | TTATTTAAGAGTAAC | 85% |
| *Streptococcus_agalactiae*_2603V/R_(AE009948) | TTATTTAAGAGTAAC | 84% |
| *Streptococcus_mutans*_GS-5_(NC_018089.1) | TTATTTAAGAGTAAC | 84% |
| *Streptococcus_pasteurianus_*ATCC43144_(NC_015600.1) | TTATTTAAGAGTAAT | 89% |
| *Streptococcus_dysgalactiae_subsp._equisimilis_*ATCC12394_(NC_017567.1) | TTATTTAAGAGTGAT | 92% |
| *Streptococcus_pyogenes_*MGAS10394_(NC_006086.1) | TTATTTAAGAGTGAT | 91% |
| *Streptococcus_uberis_*0140J_(NC_012004.1) | TTATTTAAGAGTGAT | 90% |
| *Streptococcus_equi_subsp._equi_*4047_(NC_012471.1) | TTATTTAAGAGTGAT | 87% |
| *Streptococcus_equi_subsp._zooepidemicus_*ATCC35246_(NC_017582.1) | TTATTTAAGAGTGAT | 87% |
| *Streptococcus_iniae*_SF1_(NC_021314.1) | TTATTTAAGTGTGAT | 90% |
| *Streptococcus_parauberis*_KCTC11537_(NC_015558.1) | TTATTTAAGTGTGAT | 88% |
| *Streptococcus_constellatus_subsp._pharyngis*_C1050_(NC_022238.1) | TTATTTCAAAGTAAC | 89% |
| *Streptococcus_gordonii_str._Challis_substr._*CH1_(NC_009785.1) | TTACTTAAGAGTAAC | 91% |
| *Enterococcus_faecium_*DO_(NC_017960.1) | TTATTTAACAGTTAC | 78% |
| *Enterococcus_faecalis*_62_(NC_017312.1) | TTATTTTACTGTTAC | 73% |
| *Escherichia_coli_str._K-12_substr.*_MG1655_(NC_000913.3) | TTATTTAACTTCAAC | 72% |
| *Campylobacter_jejuni_subsp._jejuni*_NCTC11168_(NC_002163.1) | TTACTTAAGTTCTAC | 66% |

Table S3 Identity of the *rplL* genes and *att*B site of ICE*Sa*2603 family in *Streptococcus* spp. ICEs were inserted in the strains highlighted in yellow background and analyzed in this study.

Table S4 MICs of the donor, recipient and conjugant strains

| Antibiotics | *S. suis* (μg/mL) | | |
| --- | --- | --- | --- |
|  | HB1011 (donor) | BAA-853 (recipient) | JH-1 (transconjugant) |
| Erythromycin | 128 | 0.125 | 64 |
| Tetracycline | 64 | 0.25 | 64 |
| Rifampicin | 0.5 | 64 | 64 |


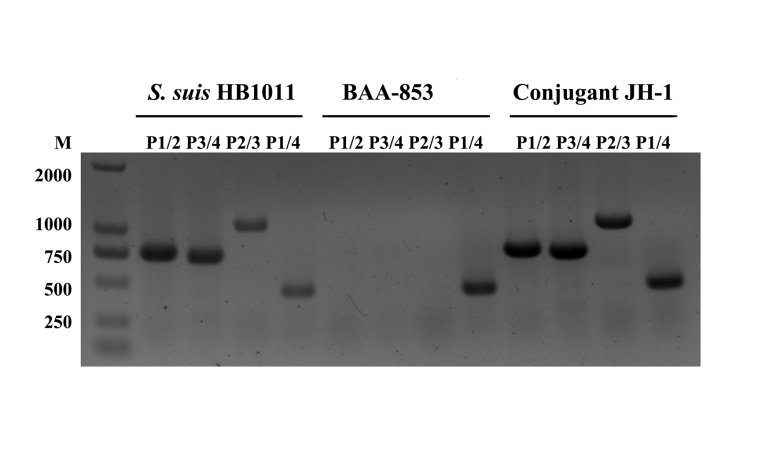


Figure S1 PCR detection of excised and circular extrachromosomal forms, as well as integrated form of the *S. suis* HB1011 (donor), BAA-853 (recipient) and JH-1 (transconjugant). The location and orientation of primers used were indicated by thin arrows shown in Figures 1B-1D.


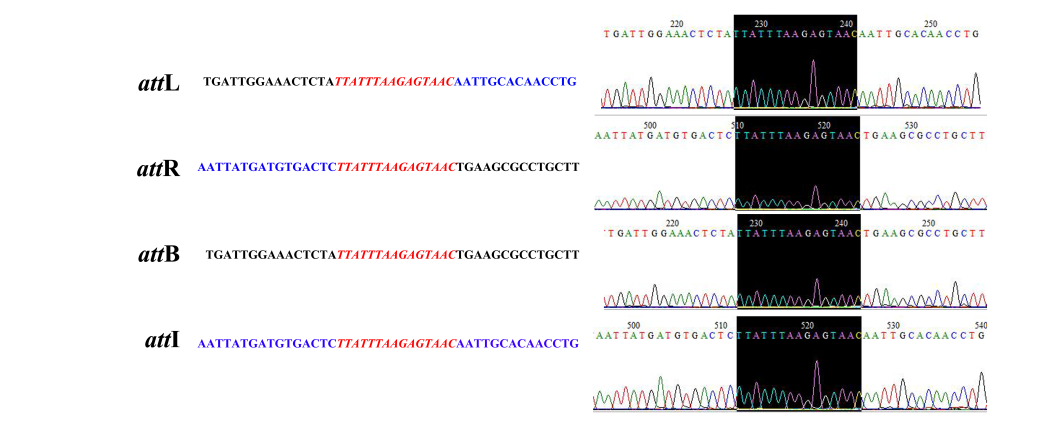


Figure S2 Sequences of the *S.suis* conjugant JH-1
